# Hydralazine-Induced Vasculitis: An Unusual Presentation of Drug-Induced Antineutrophilic Cytoplasmic Autoantibody-Associated Vasculitis

**DOI:** 10.7759/cureus.76967

**Published:** 2025-01-05

**Authors:** Sina Hedayatpour, Prakhar Singal, Kyle Madison

**Affiliations:** 1 Internal Medicine, Methodist Health System, Dallas, USA; 2 Internal Medicine, Methodist Dallas Medical Center, Dallas, USA

**Keywords:** adult pulmonology, adult rheumatology, alveolar hemorrhage, antineutrophil cytoplasmic antibody (anca) associated vasculitis (aav), drug-induced cutaneous vasculitis, • hydralazine induced anca vasculitis

## Abstract

Hydralazine is a commonly used blood pressure medication that has been associated with rheumatologic manifestations such as drug-induced lupus. However, it is rarely associated with antineutrophilic cytoplasmic autoantibody (ANCA)-associated vasculitis (AAV). Here we present a case of a 72-year-old female with hemoptysis, dyspnea, and palpable purpura, ultimately diagnosed with hydralazine-induced AAV. The diagnosis was proven via skin biopsy and serologic markers. A bronchoscopy revealed an alveolar hemorrhage. Treatment involved discontinuing hydralazine as well as steroids and rituximab. This case was atypical due to the relative lack of renal involvement. We also emphasize the importance of recognizing this rare diagnosis and discuss guideline-directed medical therapy for managing this condition.

## Introduction

Hydralazine is a commonly prescribed medication used to treat hypertension and heart failure. It acts as a smooth muscle vasodilator and is generally considered to be a safe and effective drug. However, hydralazine has been associated with several autoimmune conditions, including drug-induced lupus, cutaneous leukocytoclastic vasculitis, and rarely antineutrophilic cytoplasmic autoantibody (ANCA)-associated vasculitis (AAV) [1]. The pathogenesis of hydralazine-induced AAV is unknown but may involve cell apoptosis and the release of cytotoxic products. These products are then presented to antigen-presenting cells, triggering the production of ANCA and anti-nuclear antibodies [2,3]. Common patient characteristics associated with hydralazine-induced AAV include female sex, advanced age, and hydralazine use for at least 12 months [1]. Clinical presentations of hydralazine-induced AAV often include dyspnea, cough, fatigue, unintentional weight loss, polyarthralgia, and lower extremity petechiae [1]. Here, we present an atypical case of a patient with hemoptysis and lower extremity purpura who was diagnosed with hydralazine-induced AAV.

## Case presentation

**Figure 1:**
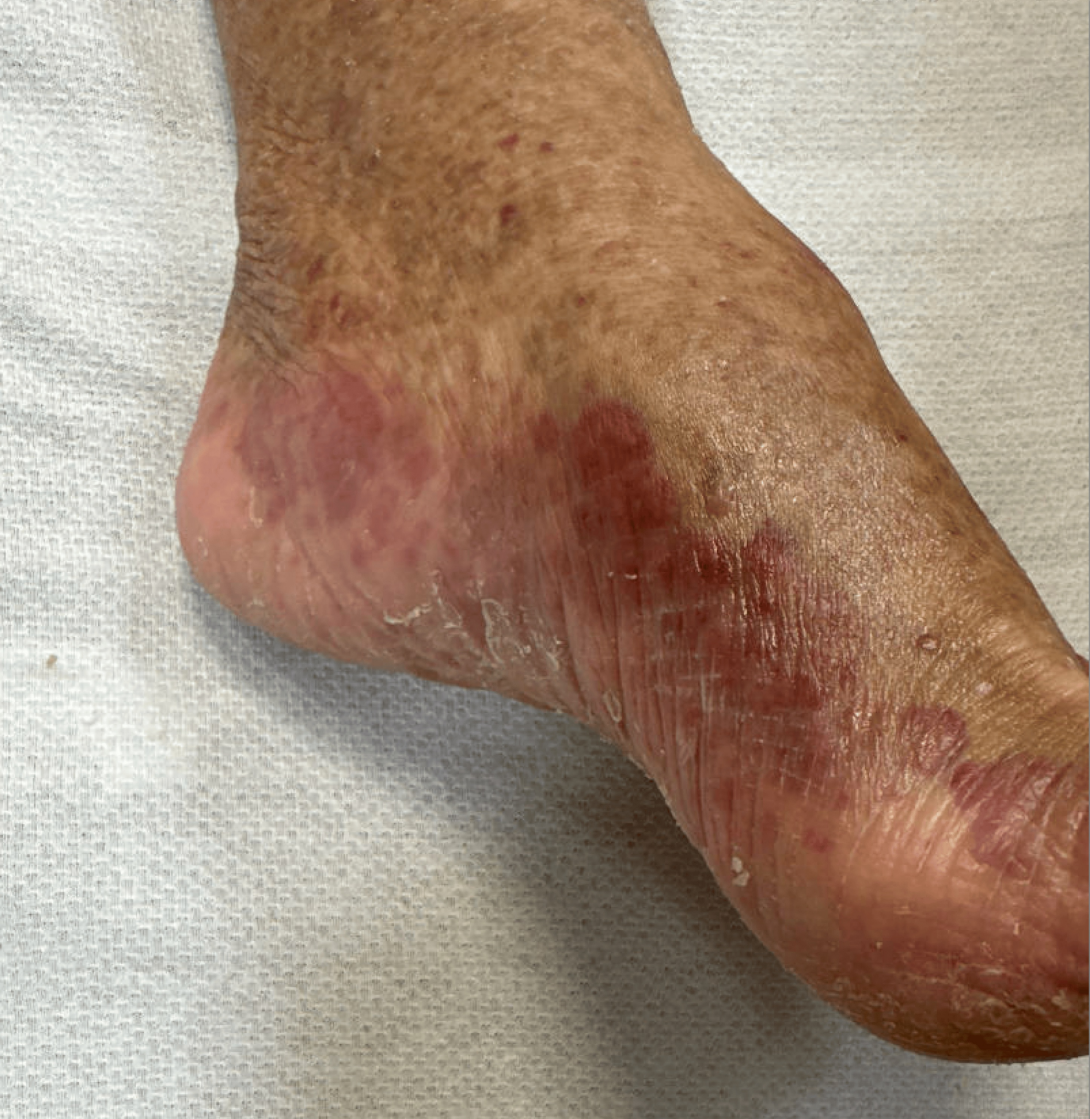
Clinical appearance of palpable purpura on the patient's left lower extremity

A 72-year-old female with a past medical history of cirrhosis secondary to non-alcoholic steatohepatitis, hypertension, and Hashimoto’s thyroiditis presented with persistent hemoptysis for approximately two weeks. Her symptoms were preceded by a three- to four-month history of generalized malaise, headache, fever, sore throat, and oral ulcers, initially attributed to a sinus infection and bronchitis. Over the past one to two months, she developed progressive dyspnea to the point where she required home oxygen. The patient initially presented to an emergency department with hemoptysis and lower extremity rashes, but elected outpatient management. However, due to worsening symptoms, she returned for further evaluation. On presentation, her vital signs included a temperature of 36.6°C, blood pressure of 134/86 mmHg, heart rate of 85 bpm, respiratory rate of 18 breaths/min, and oxygen saturation of 96% on room air. Physical examination was notable for coarse breath sounds bilaterally and palpable purpura of the lower extremities (Figure 1).

**Figure 2:**
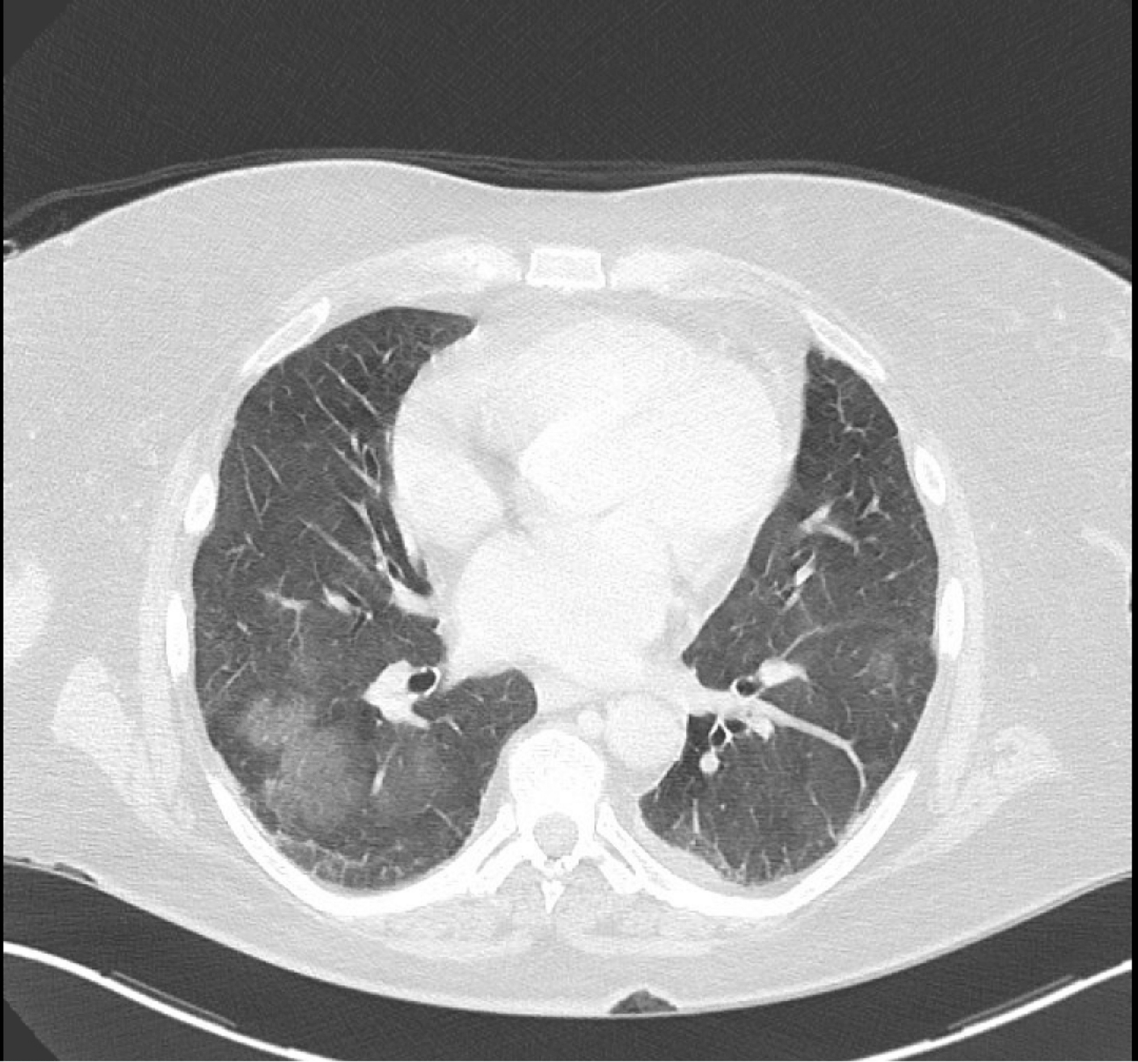
A computed tomography scan of the patient’s chest depicting bilateral central ground-glass opacities

Initial laboratory findings were notable for anemia, thrombocytopenia, an elevated sedimentation rate, and an elevated C-reactive protein. The hepatitis panel and HIV testing were negative. Urinalysis was significant for hematuria with large amounts of red blood cells (RBCs) in the urine (Table 1). A chest X-ray on admission was unremarkable, but a follow-up chest computed tomography scan revealed bilateral central ground glass opacities in the lungs (Figure 2), consistent with alveolar hemorrhage versus an infectious or inflammatory process. The initial rheumatologic workup showed negative anti-nuclear antibody and rheumatoid factors. Complement levels (C3 and C4) were at the lower limits of normal. Further autoimmune workup revealed positive ANCA titer, myeloperoxidase (MPO) antibody IgG, proteinase 3 (PR3) antibody IgG, and histone antibody IgG (Table 1). 

**Table 1:** Pertinent laboratory tests obtained with values and reference ranges Hgb: hemoglobin; Plt: platelet count; ESR: erythrocyte sedimentation rate; CRP: C-reactive protein; Ab: antibody; Ag: antigen; ANA: anti-nuclear antibody; RF: rheumatoid factor; ANCA: anti-neutrophil cytoplasmic antibody; p-ANCA: perinuclear staining; MPO: myeloperoxidase antibody; PR3: proteinase 3 antibody; IFA: immunofluorescence assay; IgG: immunoglobulin G; IgM: immunoglobulin M

Laboratory test	Value	Reference range
Hgb	8.5	Female: 12-16 g/dL
Plt	111x10^3^	150-350x10^3^ cells/mm^3^
ESR	45	Female: 0-20 mm/hr
CRP	11	0.08-3.1 mg/L
Hepatitis A Ab, IgM; Hepatitis B surface Ag; Hepatitis B core Ab, IgM; Hepatitis C Ab	Negative	Negative
HIV-1/HIV-2 Ag/Ab combo immunoassay	Negative	Negative
Urinalysis	Large urine blood; 17 RBCs/HPF	Negative blood; female: 0-4 RBCs/HPF
ANA	1.03	0.00-<1.10 index value
RF	<8.6	≤12.0 IU/mL
C3 complement	90	88-165 mg/dL
C4 complement	16	14-44 mg/dL
ANCA IFA titer	>1:1280	<1:20
ANCA IFA pattern	p-ANCA	None detected
MPO IgG	6.17	0.00-<1.10 index value
PR3 IgG	4.53	0.00-<1.10 index value
Histone IgG	6.5	0.0-0.9 units

Due to persistent hemoptysis, the patient underwent bronchoscopy, which revealed blood in the bronchi with no obvious localized source of bleeding. Sequential bronchoalveolar lavage did not show sequential progressive bloodiness, making diffuse alveolar hemorrhage less likely. Other than RBCs, the cell count and cultures were negative from the bronchoalveolar lavage. Ultimately, the patient was diagnosed with AAV in the setting of hydralazine use. Due to the worsening hemoptysis and increased oxygen requirements, the patient was initiated on pulse-dose IV methylprednisolone (1000 mg daily for five days) and a single rituximab IV infusion (1000 mg). The patient’s condition significantly improved, including decreased hemoptysis, improved lower extremity rash, and reduced oxygen requirements. At discharge, the patient was given a prednisolone taper starting at 60 mg daily, prophylaxis for *Pneumocystis jirovecii* pneumonia, instructions to follow up with rheumatology for further monitoring, and an additional outpatient rituximab infusion.

## Discussion

The patient’s clinical presentation was notable for many of the typical manifestations of vasculitis, including generalized malaise, fatigue, recurrent fever, and worsening arthralgia over several months. More significant manifestations included mild hemoptysis and a bilateral purpuric rash on the feet. Although the patient had been treated with multiple courses of antibiotics and steroids, as well as initiation of supplemental oxygen, her symptoms showed no improvement. Interestingly, the patient exhibited no signs of kidney involvement during admission. Although the lack of kidney involvement at initial presentation has been observed previously in cases of vasculitis, hydralazine-induced AAV is more frequently associated with kidney involvement [4,5]. There was initial doubt about the possibility of vasculitis during evaluation due to the absence of kidney involvement. However, the patient did develop hematuria a few weeks into hospitalization, highlighting the importance of considering vasculitis in the differential, even in the absence of kidney manifestations at presentation. This case underscores the need for careful monitoring of kidney disease development upon a diagnosis of vasculitis.

Currently, there are no universally accepted diagnostic criteria for AAV, let alone for drug-induced AAV. In this case, as there was cutaneous involvement, a skin biopsy was obtained, which revealed variable fibrinoid vascular changes affecting small vessels with surrounding neutrophilic inflammation. Pathological findings favored a diagnosis of leukocytoclastic vasculitis. Together with the positive ANCA lab serologies, the team was able to confidently make a diagnosis of AAV. Notably, the patient had dual positivity for MPO-ANCA and PR3-ANCA, a rare finding that raised suspicion of drug-induced vasculitis, consistent with findings from prior retrospective studies [6]. Hydralazine is a well-established cause of drug-induced AAV [7]. Although the patient had a normal anti-nuclear antibody level, her histone antibodies were positive, which is often observed in cases of hydralazine-induced AAV [8]. Taken together, the clinical, serologic, and histopathologic evidence strongly pointed to a diagnosis of hydralazine-induced AAV.

As the patient had significant cutaneous and pulmonary involvement, immunosuppressive treatment was deemed appropriate and initiated along with cessation of hydralazine. While no specific guidelines exist for drug-induced AAV, current American College of Rheumatology (ACR) guidelines for managing granulomatous polyangiitis or microscopic polyangiitis are available [9]. As such, these established guidelines were used as a framework for the treatment plan. Initial treatment was discontinuing the offending agent in addition to glucocorticoids. As the patient was stable with minimal hemoptysis, more cytotoxic immunosuppressive therapy was deferred. Per the 2021 ACR guidelines for the management of AAV, a reduced-dose steroid protocol was utilized after the use of IV methylprednisolone for pulse dosing [9]. For this patient, a weight-based taper with oral prednisone 60 mg daily after pulse steroids was indicated. An equivalent dose treatment of prednisolone was prescribed due to the patient’s hepatic dysfunction.

As the hospitalization progressed, the patient experienced worsening hemoptysis, classifying her disease as active severe vasculitis per ACR guidelines. Consequently, rituximab was initiated after stabilization, as it is recommended over cyclophosphamide for severe AAV per current ACR guidelines [9]. The team also considered plasma exchange as the patient’s disease severity increased. However, based on findings from the PEXIVAS trial, plasma exchange was not pursued, as it has not been shown to reduce mortality or prevent end-stage kidney disease compared to standard care [10]. Even without specific guidelines for drug-induced vasculitis, the use of other published guidelines and extrapolating them to this case provided a usable roadmap to provide suitable treatment.

## Conclusions

Providers should be aware of the possible diagnosis of hydralazine-induced AAV, as prompt discontinuation of the inciting drug is critical. Importantly, clinical manifestations do not necessarily involve the kidneys at initial presentation but may occur as the disease progresses. Without set diagnostic criteria, the use of both pathology and antibody serologies often provides enough confidence for diagnosis. For treatment, discontinuation of the offending agent alone does not appear to result in disease remission. Providers should be aware of the roles of steroids, induction agents such as rituximab, and plasma exchange in severe cases. As more cases of hydralazine-induced AAV arise, further research is essential to better understand this condition. Current guidelines primarily reflect the diagnosis and treatment of other ANCA vasculitides, highlighting the need for tailored recommendations specific to drug-induced AAV.
